# Inflammation is a target of medical treatment for lower urinary tract symptoms associated with benign prostatic hyperplasia

**DOI:** 10.1007/s00345-020-03106-1

**Published:** 2020-02-14

**Authors:** Cosimo De Nunzio, Andrea Salonia, Mauro Gacci, Vincenzo Ficarra

**Affiliations:** 1 Department of Urology, Sant’Andrea Hospital, Sapienza University of Rome, Rome, Italy; 2 University Vita-Salute San Raffaele, Milan, Italy; 3 Division of Experimental Oncology/Unit of Urology, URI, IRCCS Ospedale San Raffaele, Milan, Italy; 4 Minimally Invasive and Robotic Surgery, and Kidney Transplantation, University of Florence AOUC-Careggi Hospital, Florence, Italy; 5 Department of Human and Pediatric Pathology “Gaetano Barresi”, Urologic Section, University of Messina, Piazza Pugliatti, 1, 98122 Messina, ME Italy

**Keywords:** Prostatic hyperplasia, Prostatic inflammation, Progression, Medical therapy, Phytotherapy, *Serenoa repens*

## Abstract

**Purpose:**

To review the role of a persistent prostatic inflammatory status (PIS) in the development and progression of benign prostatic hyperplasia (BPH) associated with lower urinary tract symptoms (LUTS) and which medical therapies approved for LUTS/BPH may reduce persistent PIS.

**Methods:**

Literature search in PubMed up to July 2019.

**Results:**

The cause of histologically defined persistent PIS or chronic prostatic inflammation is multifactorial. It is evident in many men with LUTS/BPH, particularly in older men and in men with a large prostate volume or more severe (storage) LUTS. Additionally, persistent PIS is associated with an increased risk of acute urinary retention and symptom worsening. Of medical therapies approved for LUTS/BPH, the current evidence for a reduction of persistent PIS is greatest for the hexanic extract of *Serenoa repens* (HESr). This treatment relieves LUTS to the same extent as α_1_-adrenoceptor antagonists and short-term 5α-reductase inhibitors. Limited evidence is available on the effect of other mainstream LUTS/BPH treatments on persistent PIS.

**Conclusions:**

Persistent PIS plays a central role in both the development and progression of LUTS/BPH. In men with LUTS/BPH who have a high chance of harbouring persistent PIS, HESr will not only improve LUTS, but also reduce (underlying) inflammation. Well-designed clinical studies, with a good level of evidence, are required to better evaluate the impact of BPH/LUTS medical therapies on persistent PIS.

**Electronic supplementary material:**

The online version of this article (10.1007/s00345-020-03106-1) contains supplementary material, which is available to authorized users.

## Introduction

Benign prostatic hyperplasia (BPH) is the most common, chronic, slowly progressing urological disease in elderly men, evident in 50% of men in their 50 s and in 90% in their 80 s [1]. Clinically, it can be associated with benign prostatic enlargement (BPE) and eventually benign prostatic obstruction (BPO), causing bladder outlet obstruction (BOO) along with lower urinary tract symptoms (LUTS) [2]. Among LUTS, it is possible to define symptoms related to the storage and/or the voiding phase of micturition. LUTS have a significant effect on patients’ quality of life.

The pathogenesis and progression of BPH is still not fully understood but is most likely multifactorial with increased sympathetic nervous activity, hormonal alterations, the presence of the metabolic syndrome (MetS) [3] and tissue remodelling related to ageing playing a role. In the last decades, growing interest exists for the hypothesis that BPH is an immune-mediated inflammatory disease with a persistent prostatic inflammatory status (PIS) as a key factor throughout the development and progression of BPH [2]. This review provides the latest update on how persistent PIS and BPH/LUTS are interrelated and the potential impact of LUTS/BPH medical treatment on persistent PIS.

## Methods

A non-systematic review of the literature for English-language original and review articles (e)-published up to July 2019 (no date restriction) was performed using the National library of Medicine’s PubMed database. The used search strategy included the following terms and limits:

- (Prostatic hyperplasia (Mesh)) AND (Prostatitis (Mesh) OR inflammation), limited for English language, Abstract available, Humans and Title
- “(prostate OR prostatic) inflammation” AND (serenoa OR alfuzosin OR doxazosin OR naftopidil OR silodosin OR tamsulosin OR terazosin OR dutasteride OR finasteride OR tadalafil)
- “serenoa repens” AND (“benign prostatic hyperplasia” OR “BPH”)
- “serenoa repens” AND (“benign prostatic hyperplasia” OR “BPH”) AND (alfuzosin OR doxazosin OR naftopidil OR silodosin OR tamsulosin OR terazosin OR dutasteride OR finasteride OR tadalafil)

The abstracts of the retrieved records (maximum *N* = 707 due to overlap between the papers retrieved from the different searches, Online Resource 1) were screened by three of the authors to identify and read the most relevant articles. Additionally, other significant studies cited in the reference lists of the selected papers were evaluated. Studies published only as abstracts and meeting reports were not included in the review. Being a non-systematic review, the selection of references was by definition not all-inclusive and selection bias may have occurred.

### Persistent prostatic inflammation: definition and etiopathogenesis

**Fig. 1:**
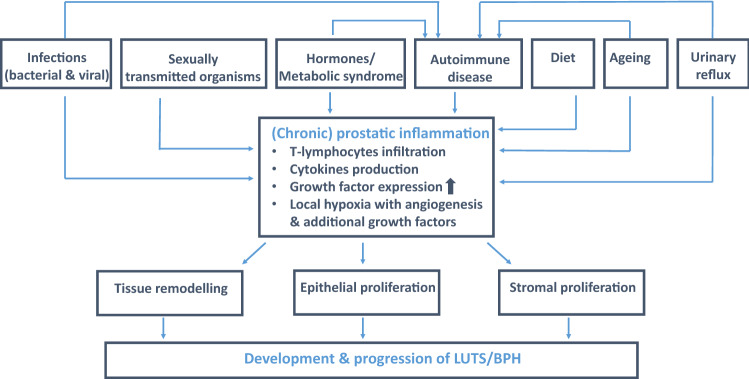
Hypothesis on how persistent PIS can develop and how this may contribute to the development and progression of BPH (modified from [5])

An inflammatory reaction in prostatic tissue can be triggered by several factors, including bacterial infections, viruses (e.g., human papilloma virus, herpes simplex virus type 2, and cytomegalovirus), sexually transmitted organisms (e.g., gonorrhoeae and chlamydia), hormones, the MetS, dietary factors, urinary reflux as well as an autoimmune response (Fig. 1) [4–8]. The infiltrating inflammatory cells (70–80% T-lymphocytes, 10–15% B-lymphocytes and 15% macrophages) become activated and release pro-inflammatory cytokines, which in turn increase the expression of several growth factors (e.g., interleukin (IL)-17, IL-15, IL-8, interferon-γ, fibroblast growth factor [FGF] and FGF-2), resulting in abnormal proliferation of the epithelial and stromal cells. The subsequent increased oxygen demand of these cells leads to local hypoxia producing low levels of reactive oxygen species (ROS) promoting angiogenesis and the production of additional growth factors (i.e., vascular endothelial growth factor, IL-8, FGF-2, FGF-7 and transforming growth factor ß) [4]. As such, persistent PIS or chronic prostatic inflammation is a histological observation and, irrespective of the mechanism that triggers the uncontrolled inflammatory response, the final result of this process induces tissue damage with subsequent abnormal wound healing and stromal and epithelial cell proliferation and thus BPH [4]. In basic science literature, inflammation is usually described in terms of cellular effectors and released mediators. Several authors have used the score proposed by Irani et al. [9] to classify PIS. This score classifies prostatic inflammation based on the histological grading for the extension of inflammatory cells (from grade 0, no inflammatory cells, to grade 3, large inflammatory areas) and for the effect of these cells on prostatic tissue (i.e., aggressiveness grading ranging from grade 0, no contact between inflammatory cells and glandular epithelium, to grade 3, > 25% glandular disruption). The infiltration of inflammatory cells in the prostate during the development of BPH should be differentiated from classical chronic prostatitis, which is related to chronic pelvic pain.

### Persistent prostatic inflammation and BPH development/progression

Inflammatory infiltrates have been demonstrated in biopsy samples and surgical specimens of prostatic tissue of patients with LUTS/BPH [4–6]. In 3942 surgery-derived BPH specimens, inflammation was observed in 43%, of which 69% concerned chronic inflammation [10]. The inflammation was significantly associated with age and prostate volume (PV): 61% of prostates 80–89 mL had chronic inflammation versus 8.5% of those 30–39 mL. In 1198 patients with LUTS/BPH from the Medical Therapy of Prostate Symptoms (MTOPS) study, approximately 40% of the baseline prostate biopsy specimens showed chronic inflammatory infiltrates (especially in older men and men with a larger PV) [11]. In 8224 LUTS/BPH patients from the Reduction by Dutasteride of prostate Cancer Events (REDUCE) study, 77.6% of the prostate biopsy samples contained chronic inflammatory cells/persistent PIS. A weak but statistically significant correlation between persistent PIS and an increased PV and the International Prostate Symptom Score (IPSS), particularly the storage subscore, was observed [12]. An autopsy study of 320 prostate glands from men aged 30–80 years showed persistent PIS in 74.5% of the specimens [13]. Men with inflammation were 6.8 times more likely to have BPH than men without inflammation. Furthermore, a study in 282 LUTS/BPH patients undergoing surgery showed persistent PIS in 79%, 48%, and 20% of patients with severe, intermediate, and no LUTS/BPH, respectively [14, 15]. Mean PV was statistically significantly higher in those with high-grade versus those with low-grade inflammation (77 versus 62 mL). The same applied for the mean total IPSS (21 versus 12 points). Other studies have also confirmed that persistent PIS is associated with PV and (storage) symptom severity in men with LUTS/BPH [16].

The impact of persistent prostatic inflammation on BPH progression has also been investigated. In the placebo group of the MTOPS study, LUTS/BPH patients with persistent PIS were at increased risk of developing acute urinary retention (AUR), symptom worsening and the need for BPH-related surgery as compared to those without inflammation [11]. The risk of AUR was 5.6% versus 0% (*P* = 0.003), respectively. Also LUTS/BPH patients in the placebo group of the REDUCE study (*N* = 4109) who had persistent PIS at baseline were at increased risk of developing AUR (hazard ratio 1.6–1.8, *P* = 0.001) [17]. However, persistent PIS was not associated with symptom worsening over time (median follow-up 41.4 months). Only in patients with a moderate-marked persistent PIS at baseline, a weak association with LUTS/BPH progression was found in post-hoc analyses [17]. The difference between the results from the MTOPS and REDUCE study in terms of symptom progression may have been due to the fact that the REDUCE study included older men and excluded patients with severe BPH (PV > 80 mL and total IPSS > 25 or > 20 while on α_1_-adrenoceptor antagonist treatment) at baseline. Several older, smaller scale studies have confirmed that persistent PIS is not only associated with the development but also with the (faster) progression of BPH [18–20].

### Persistent prostatic inflammation and LUTS/BPH: implication for diagnosis and treatment

Until now, histological examination of prostate biopsies remains the only available method to show the presence of inflammatory prostatic cells. As this is feasible only in patients with suspicious prostate cancer, less invasive tools are needed for identifying patients who are at high risk of harbouring persistent PIS. As discussed, patients with severe LUTS (a high total IPSS, e.g., ≥ 20 points), particularly those with storage symptoms, are at increased risk of having persistent PIS [7].

Also those with prostatitis-like symptoms, such as pain and burning sensation, dribbling and hesitant urination, urgency, pain or discomfort of the penis and testicles and painful ejaculations, may have inflammation upon biopsy as shown in another sub-analysis of the REDUCE study [7, 21].

Prostatic calcifications identified at ultrasonography may also provide a hint for the presence of persistent PIS in men with LUTS/BPH. Prostatic calcifications can produce obstruction of the intraprostatic ducts. This stimulates an inflammatory response, characterised by lymphocyte and cytokine activation and ROS release, with subsequent tissue damage and wound healing with stromal proliferation and excessive extracellular matrix production [7, 22].

Additionally, serum/plasma or urine biomarkers could be used to identify LUTS/BPH patients who have a high chance of having persistent PIS [23]. Seminal plasma IL-8 levels [7] are the most reliable and predictive surrogate marker for diagnosing persistent PIS [24, 25] and have been shown to be significantly higher in patients with both LUTS/BPH and chronic prostatitis than in patients with LUTS/BPH only [26]. Unfortunately, the use of this biomarker is expensive and not popular and will therefore probably require further clinical evaluation before it can be introduced in routine clinical practice [22]. Other potential biomarkers [7, 8], still under investigation, include serum C-reactive protein (CRP), monocyte chemotactic protein-1 (MCP-1) in prostatic secretions and chemokine (C–C motif) receptor 7 (CCR7), cytotoxic T-lymphocyte-associated antigen (CTLA4), inducible T cell costimulatory (ICOS), and CD40 ligand (CD40LG) in urine.

Finally, as persistent PIS seems to be the link between the MetS and BPH and MetS patients have increased levels of CRP, several ILs and tumour necrosis factor (TNF)-α, also obese men with LUTS/BPH who have an increased insulin resistance, hypertension and hypercholesterolaemia may harbour persistent PIS [3, 7].

Several drug classes are approved and strongly recommended by the European Association of Urology (EAU) guidelines for the treatment of LUTS/BPH [2]. These include α_1_-adrenoceptor antagonists, 5α-reductase inhibitors (5ARIs) and phosphodiesterase type 5 inhibitors (PDE5I). Moreover, although not approved for LUTS/BPH by health authorities, muscarinic receptor antagonists and beta-3 agonists are recommended by the EAU guidelines for men with moderate-to-severe LUTS who have mainly bladder storage symptoms [2]. The use of a particular drug class or the combination of different drug classes depends on, e.g., the type of LUTS, LUTS severity, PV, treatment duration, risk of progression and patient preference [2]. Although current data don’t allow the EAU guidelines to make any recommendations about plant extracts for the treatment of BPH/LUTS, long-term clinical experience exists with plant extracts. The main plant extracts are *Cucurbita pepo* (pumpkin seeds), *Hypoxis rooperi* (South African star grass), *Pygeum africanum* (bark of the African plum tree), *Secale cereal* (rye pollen), *Serenoa repens* (syn. Sabal serrulata; saw palmetto) and *Urtica dioica* (roots of the stinging nettle) [2]. The most widely used [27, 28] and also most thoroughly studied plant extract in basic and clinical research for LUTS/BPH is *Serenoa repens* [28]. As the different extracts of the same plant available on the market do not necessarily have the same biological or clinical effects (e.g., due to the use of different extraction processes), the effects of one brand cannot be extrapolated to others [2, 29, 30]. A Cochrane meta-analysis published in 2012, including 5666 men (32 randomised controlled trials [RCTs] with trial lengths of 4–72 weeks), found no difference between *Serenoa repens* and placebo in changes in symptom scores [31]. However, this review combined data from various brands and the authors acknowledged that their findings may not be generalised to proprietary products [29]. Two more recent meta-analyses focussing on the hexanic extract of *Serenoa repens* (HESr) found that treatment with this extract reduced nocturia and improved maximum urinary flow rate (*Q*_max_) compared to placebo and had similar efficacy to tamsulosin and short-term 5ARIs for relieving LUTS. The European Medicines Agency (EMA) has only granted HESr as a well-established medicinal product since the use of this plant extract is supported by sufficient evidence of efficacy and safety [29, 32].

There is very limited data on the impact of α_1_-adrenoceptor antagonists and 5ARIs on persistent PIS. In a rat model of urine reflux-induced prostatic inflammation, silodosin prevented the upregulation of inflammation-associated proteins (IL-1α, IL-1ß, IL-6, and TNF-α) and prostatic hypoxia, which was attributed to an improvement of prostate blood flow [33]. In a rat model of chronic bacterial prostatitis, finasteride statistically significantly decreased bacterial growth and reduced inflammatory cell infiltrations [34]. In a mouse xenograft model of human BPH, dutasteride reduced staining of cyclooxygenase-2 (Cox-2) and Ras homolog gene family, member A (RhoA) after already 2 months [5, 35]. In contrast, another study in a mouse model of BPH showed that finasteride increased persistent PIS (i.e., CD45 + cell foci) [36]. In a study of 17 patients with BPH undergoing transurethral resection of the prostate (TURP), doxazosin (*N* = 4) reduced the staining of CD3 and CD68 compared to no treatment (*N* = 5) [37]. Glutathion-S-transferase pi-1 (GSTP1: inactivates oxidant carcinogens) expression was only decreased in patients receiving both doxazosin and finasteride (*N* = 8) suggesting that finasteride may interfere with the anti-inflammatory effect of doxazosin. A retrospective Korean study in 82 patients with BPH confirmed on biopsy and treated for 12 months with α_1_-adrenoceptor antagonists and 5ARIs showed reduced improvement in storage symptoms from 3 months onwards in those patients having high-grade chronic inflammation (based on the Irani grading system; *N* = 44), whereas this did not occur in the low-grade group (*N* = 38) [38]. Although this difference was not statistically significant, the patients in the high-grade group also more frequently needed surgery (9.1% of patients) because of AUR or insufficient therapeutic response compared to those in the low-grade group (0%). This suggests that LUTS/BPH patients harbouring persistent PIS may fail treatment with α_1_-adrenoceptor antagonists and 5ARIs in the long-term [5, 7, 8]. However, this study did not distinguish between the effect of α_1_-adrenoceptor antagonists and 5ARIs. Another retrospective Korean study in 111 LUTS/BPH patients treated with the α_1_-adrenoceptor antagonist tamsulosin 0.2 mg/day for only 3 months indicated that the improvement in LUTS was independent of the inflammation grade [39]. However, multivariate analysis suggested that longer duration of treatment was associated with decreased symptomatic improvement (odds ratio 0.92; 95% CI 0.85–0.99). This suggests that response to treatment with α_1_-adrenoceptor antagonists is influenced by the presence of persistent PIS but that at the same time it can modulate prostate immune cell activity [5]. The effect of both α_1_-adrenoceptor antagonists and 5ARIs on persistent PIS obviously needs further (clinical) investigation.

Regarding the PDE5I tadalafil, two animal studies have shown anti-inflammatory effects [40–42]. Moreover, in human BPH stromal cell lines, tadalafil blunted IL-8 secretion induced by metabolic stimuli or TNF-α [5, 40, 43]. In tissue of men with LUTS/BPH under low androgen conditions (i.e., treated with finasteride), tadalafil was able to reduce T-cell infiltration and related CCL5 secretion resulting in decreased proliferation of BPH epithelial cells [44].

**Fig. 2:**
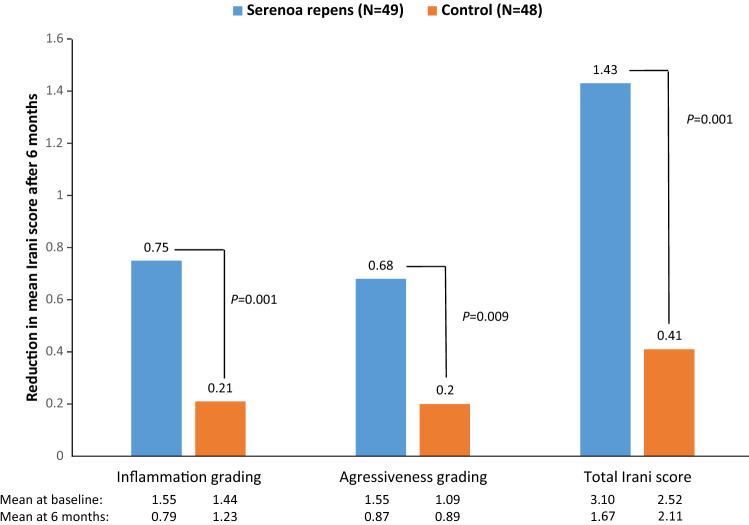
Histopathological findings according to Irani’s score at baseline (prostate biopsy 1) and after 6 months (prostate biopsy 2) [55]. The Irani score classifies prostatic inflammation on the basis of the extension of inflammatory cells and their effect on prostate tissue. A four-point scale is used for inflammation (0: no inflammatory cells, 1: scattered inflammatory cell infiltrate, 2: nonconfluent lymphoid nodules, 3: large inflammatory areas with confluence of infiltrate) and aggressiveness (0: no contact between inflammatory cells and glandular epithelium, 1: contact between inflammatory cell infiltrate and glandular epithelium, 2: clear but limited, i.e., < 25% of the examined material, shows glandular epithelium disruption, 3: glandular epithelium disruption in > 25% of the examined material)

Several in vitro and in vivo studies [45] have shown that HESr demonstrates inhibition of both inflammatory cells [46, 47] and a wide variety of inflammatory mediators and proteins [47–52], as well as deregulation of numerous genes playing a role in the proliferative, apoptotic, and inflammatory pathways of BPH itself [53]. In a double-blind, randomised study involving 206 LUTS/BPH patients treated with HESr (320 mg/day) or tamsulosin (0.4 mg/day) for 3 months, HESr reduced mean mRNA expression of the 15 out of 29 most frequently expressed inflammation-related genes in urine in 80% versus 33% of the genes with tamsulosin [54]. In addition, the macrophage migration inhibitory factor (MIF) was downregulated in a higher proportion of HESr (42.5%) as compared to tamsulosin-treated patients (23.9%) and upregulated in a lower proportion (43.8% versus 66.2%). Moreover, in contrast to tamsulosin, the reduction in mean total IPSS with HESr was larger in patients with MIF overexpression at baseline (6.4 points) versus patients without MIF overexpression at baseline (4.5 points). In line with these anti-inflammatory effects, in a RCT of 97 patients with histologically/prostate biopsy confirmed prostatic inflammation, 6 months of treatment with HESr (320 mg/day) improved Irani’s inflammation grading, aggressiveness, and total score in the biopsy to a statistically significant greater extent than in control patients who received no treatment (Fig. 2) [55]. Moreover, the inflammation score was upgraded in 25% of patients in the control group versus 6.1% of patients in the HESr group. Likewise, HESr also statistically significantly improved the immunohistological staining of antibodies against T and B-lymphocytes as well as macrophages. Therefore, in patients with LUTS/BPH, HESr may not only relieve LUTS to the same extent as α_1_-adrenoceptor antagonists and short-term 5ARIs, but also reduce the underlying inflammation [27, 28, 54–58]. This would be particularly useful in LUTS/BPH patients who have a high chance of harbouring persistent prostatic inflammation.

**Fig. 3:**
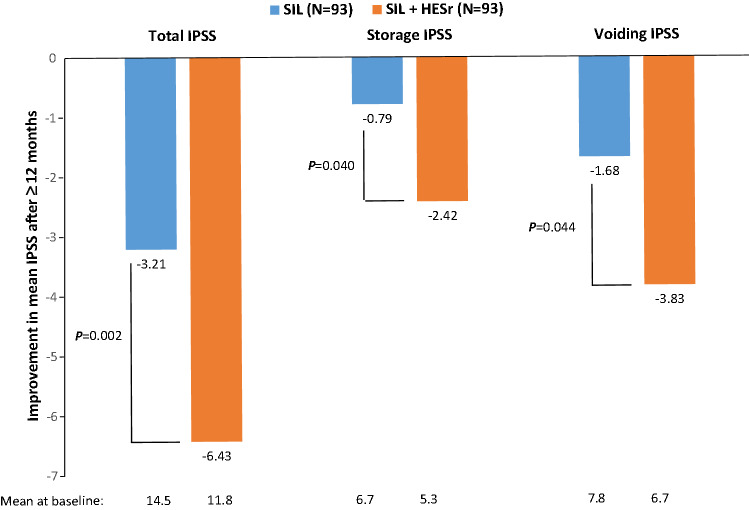
Improvement in mean IPSS after ≥ 12 months of treatment with silodosin 8 mg/day (SIL) or its combination with HESr 320 mg/day (SIL + HESr) in 186 men with LUTS/BPH [61]

The effect of combination therapy of HESr with other approved LUTS/BPH treatments on LUTS has been investigated in several studies. In a French randomised study involving 352 LUTS/BPH patients treated for 1 year, the combination of tamsulosin (0.4 mg/day) and HESr (320 mg/day) was not significantly superior to tamsulosin alone with regard to improvement of the total IPSS (6.0 versus 5.2 points, respectively; *P* = 0.286) [59]. Nevertheless, an open-label, randomised, Korean study in 103 LUTS/BPH patients showed that 1 year of treatment with the combination of tamsulosin (0.2 mg/day) and HESr (320 mg/day) was equally effective as tamsulosin monotherapy in reducing total and voiding IPSS, but resulted in a greater improvement of the storage IPSS (1.9 versus 0.9 points, *P* = 0.021) [59, 60]. In both studies, there was only a limited increase in adverse drug reactions in the combination group [59, 60], mainly gastrointestinal disorders [60]. A third Italian cross-sectional, matched-pair study compared monotherapy of silodosin (8 mg/day) to its combination with HESr in 186 LUTS/BPH patients treated for ≥ 1 year [61]. The mean improvement in total IPSS was significantly greater in patients receiving combination therapy (6.43 points) compared to those receiving silodosin alone (3.21 points, *P* = 0.002); of clinical relevance, this applied for both the voiding and storage component of the IPSS (Fig. 3). The greater improvement in *Q*_max_ with combination therapy (4.3 versus 2.3 mL/s) was not significant (*P* = 0.15). Currently, no studies are published comparing the efficacy of the combination of an 5ARI or PDE5I with HESr versus monotherapy.

## Conclusions

This extensive, non-systematic literature review indicates that persistent PIS is particularly evident in men with LUTS/BPH who are older, have a large PV, more severe (storage) LUTS and/or MetS. Persistent PIS per se contributes to the development of BPH and also increases the risk of (faster) progression. Of the medical therapies approved for the treatment of LUTS/BPH, HESr showed the greatest evidence for a reduction of persistent PIS. However, it should be noted that there is limited data on the effect of LUTS/BPH medical therapies on persistent PIS and these studies are often limited by their low level of evidence. HESr seems to relieve LUTS to the same extent as α_1_-adrenoceptor antagonists and short-term 5ARIs and to reduce the underlying inflammation. Combining HESr with an α_1_-adrenoceptor antagonist may relief storage symptoms (who are linked to persistent PIS) but also voiding symptoms to a greater extent than α_1_-adrenoceptor antagonist monotherapy. Future well-designed clinical trials, with a good level of evidence, should (better) evaluate the impact of HESr, PDE5I, α_1_-adrenoceptor antagonists and 5ARIs (alone or in combination) on persistent PIS and the potential impact of targeting the inflammatory pathway on LUTS/BPH development and progression.

## Electronic supplementary material

Below is the link to the electronic supplementary material.

Supplementary file1 (PDF 93 kb)
