# Correction to: Inflammation is a target of medical treatment for lower urinary tract symptoms associated with benign prostatic hyperplasia

**DOI:** 10.1007/s00345-020-03290-0

**Published:** 2020-06-10

**Authors:** Cosimo De Nunzio, Andrea Salonia, Mauro Gacci, Vincenzo Ficarra

**Affiliations:** 1 Department of Urology, Sant’Andrea Hospital, Sapienza University of Rome, Rome, Italy; 2 University Vita-Salute San Raffaele, Milan, Italy; 3 Division of Experimental Oncology/Unit of Urology, URI, IRCCS Ospedale San Raffaele, Milan, Italy; 4 Minimally Invasive and Robotic Surgery, and Kidney Transplantation, University of Florence AOUC-Careggi Hospital, Florence, Italy; 5 Department of Human and Pediatric Pathology “Gaetano Barresi”, Urologic Section, University of Messina, Piazza Pugliatti, 1, 98122 Messina, ME Italy

## Correction to: World Journal of Urology 10.1007/s00345-020-03106-1

The article “Inflammation is a target of medical treatment for lower urinary tract symptoms associated with benign prostatic hyperplasia”, written by Cosimo De Nunzio, Andrea Salonia, Mauro Gacci and Vincenzo Ficarra was originally published electronically on the publisher’s internet portal on February 14, 2020 without open access. With the author(s)’ decision to opt for Open Choice the copyright of the article changed on June 8, 2020 to © The Author(s) 2020 and the article is forthwith distributed under a Creative Commons Attribution 4.0 International License (https://creativecommons.org/licenses/by/4.0/), which permits use, sharing, adaptation, distribution and reproduction in any medium or format, as long as you give appropriate credit to the original author(s) and the source, provide a link to the Creative Commons licence, and indicate changes were made.

The original article has been corrected.

