# Supplementary material for: Inflammation is a target of medical treatment for lower urinary tract symptoms associated with benign prostatic hyperplasia
Source: World J Urol. 2020 Feb 14;38(11):2771–9. doi: 10.1007/s00345-020-03106-1 (PMC7644532; doi:10.1007/s00345-020-03106-1)
Supplement: Supplementary file 1 — Supplementary file1 (PDF 93 kb) [file 345_2020_3106_MOESM1_ESM.pdf]

Inflammation is a target of medical treatment for lower urinary tract symptoms associated with benign prostatic hyperplasia

World Journal of Urology

Cosimo De Nunzio[a], Andrea Salonia[b,c] Mauro Gacci[d], Vincenzo Ficarra[e]

a) Sant'Andrea Hospital, Sapienza University of Rome, Department of Urology, Rome, Italy

b) University Vita-Salute San Raffaele, Milan, Italy

c) Division of Experimental Oncology/Unit of Urology; URI; IRCCS Ospedale San Raffaele, Milan, Italy

d) Minimally Invasive and Robotic Surgery, and Kidney Transplantation, University of Florence AOUC-Careggi Hospital, Florence, Italy

e) Department of Human and Pediatric Pathology "Gaetano Barresi", Urologic Section, University of Messina, Messina, Italy

Corresponding author: Vincenzo Ficarra vficarra@unime.it

Online Resource 1: Literature searches performed in PubMed in July 2019

**Search terms**	**Limitations**	**# of records**
(Prostatic hyperplasia (Mesh)) AND (Prostatitis (Mesh) OR inflammation)	English language, Abstract available, Humans, Title	348
"(prostate OR prostatic) inflammation" AND serenoa		24
"(prostate OR prostatic) inflammation" AND tamsulosin		14
"(prostate OR prostatic) inflammation" AND alfuzosin		6
"(prostate OR prostatic) inflammation" AND doxazosin		6
"(prostate OR prostatic) inflammation" AND terazosin		6
"(prostate OR prostatic) inflammation" AND silodosin		3
"(prostate OR prostatic) inflammation" AND naftopidil		1
"(prostate OR prostatic) inflammation" AND finasteride		37
"(prostate OR prostatic) inflammation" AND dutasteride		20
"(prostate OR prostatic) inflammation" AND tadalafil		11
"serenoa repens" AND ("benign prostatic hyperplasia" OR "BPH")		157
“serenoa” AND “tamsulosin”		27
“serenoa” AND “alfuzosin”		6
“serenoa” AND “terazosin”		3
“serenoa” AND “doxazosin”		2
“serenoa” AND “silodosin”		1
“serenoa” AND “naftopidil”		0
serenoa AND finasteride AND ("benign prostatic hyperplasia" OR "BPH")		31
serenoa AND dutasteride AND ("benign prostatic hyperplasia" OR "BPH")		4
